# Florence Nightingale

**Published:** 1892-12

**Authors:** 


### FLORENCE NIGHTINGALE.

Florence Nightingale, the celebrated English philanthropist, was born in Florence, Italy, May, 1820. She was the younger daughter of William Edward Shore, a Sheffield banker, who inherited the estates of Peter Nightingale, and in accordance with the terms of the latter's will, took the surname. Florence was highly educated in the classics, mathematics, the modern languages and music; but her favorite study was the care of the sick. In 1849, she took a course of training in Pastor Fliedner's school of Deaconnesses, at Kaiserswerth. In 1851 she took charge of a sanatarium for infirm and invalid governesses, and greatly improved the management of it. In 1854, she went to the army in the Crimea as superintendent of a corps of volunteer female nurses—ninety-two in number — and organized a hospital at Scutari, on November 5. On the 7th, they received 600 soldiers, and in three weeks their number had increased to 3,000. In spite of her many discouragements Miss Nightingale made her hospital a model of thoroughness and perfection, and all other hospitals on the Bosphorus were put under her superintendence. In September, 1856, having suffered a severe attack of hospital fever, Miss Nightingale returned to England, broken down in health. She received a jewel with a letter of thanks for her noble services, and a fund of £50,000 was raised to found a school for nurses under her direction. The soldiers of the Crimean war made a penny contribution to raise a statue in her honor, but she would not permit it.

